# Association of *SLC12A1* and GLUR4 Ion Transporters with Neoadjuvant Chemoresistance in Luminal Locally Advanced Breast Cancer

**DOI:** 10.3390/ijms242216104

**Published:** 2023-11-09

**Authors:** Montserrat Justo-Garrido, Alejandro López-Saavedra, Nicolás Alcaraz, Carlo C. Cortés-González, Luis F. Oñate-Ocaña, Claudia Haydee Sarai Caro-Sánchez, Clementina Castro-Hernández, Cristian Arriaga-Canon, José Díaz-Chávez, Luis A. Herrera

**Affiliations:** 1 Cancer Research Unit, Institute of Biomedical Research, National Autonomous University of Mexico (UNAM)-National Institute of Cancerology, San Fernando Av #22, XVI Section, Mexico City 14080, Mexico; montserrat.justo@gmail.com (M.J.-G.); alexlosaav@gmail.com (A.L.-S.); cesarcortesg@gmail.com (C.C.C.-G.); ccastroh7@yahoo.com.mx (C.C.-H.); cristiancanon@hotmail.com (C.A.-C.); 2 The Bioinformatics Centre, Department of Biology, University of Copenhagen, 2200 Copenhagen, Denmark; alcaraz@cpr.ku.dk; 3 Novo Nordisk Foundation Center for Protein Research, Faculty of Health and Medical Sciences, University of Copenhagen, 1165 Copenhagen, Denmark; 4 Department of Gastroenterology, National Cancer Institute (INCan), Tlalpan, Mexico City 14080, Mexico; lfonate@gmail.com; 5 Molecular Pathology and Immunology, National Cancer Institute (INCan), Tlalpan, Mexico City 14080, Mexico; dra.haydee.caro@gmail.com; 6 School of Medicine and Health Sciences-Tecnológico de Monterrey, Mexico City 14380, Mexico

**Keywords:** luminal breast cancer, locally advanced, chemoresistance, neoadjuvant chemotherapy, ion transport

## Abstract

Chemoresistance to standard neoadjuvant treatment commonly occurs in locally advanced breast cancer, particularly in the luminal subtype, which is hormone receptor-positive and represents the most common subtype of breast cancer associated with the worst outcomes. Identifying the genes associated with chemoresistance is crucial for understanding the underlying mechanisms and discovering effective treatments. In this study, we aimed to identify genes linked to neoadjuvant chemotherapy resistance in 62 retrospectively included patients with luminal breast cancer. Whole RNA sequencing of 12 patient biopsies revealed 269 differentially expressed genes in chemoresistant patients. We further validated eight highly correlated genes associated with resistance. Among these, solute carrier family 12 member 1 (*SLC12A1*) and glutamate ionotropic AMPA type subunit 4 (*GRIA4*), both implicated in ion transport, showed the strongest association with chemoresistance. Notably, *SLC12A1* expression was downregulated, while protein levels of glutamate receptor 4 (GLUR4), encoded by *GRIA4*, were elevated in patients with a worse prognosis. Our results suggest a potential link between *SLC12A1* gene expression and GLUR4 protein levels with chemoresistance in luminal breast cancer. In particular, GLUR4 protein could serve as a potential target for drug intervention to overcome chemoresistance.

## 1. Introduction

Breast cancer remains the most prevalent cancer in women, accounting for 24.5% of all tumors found and over 2.2 million new cases in 2020 [1]. The mortality from breast cancer is higher in lower-income countries, like in Latin America, where approximately 70% of diagnoses are in patients with large-sized, node-positive, or inoperable tumors, known as locally advanced breast cancer (LABC) [2,3,4]. The primary treatment for LABC involves a neoadjuvant chemotherapy (NAC) regimen consisting of taxanes and anthracyclines [5]. NAC is administered prior to surgery and serves multiple purposes. Firstly, it helps render large tumors operable by reducing their size. Additionally, NAC allows for monitoring of treatment responses, aiding in the determination of whether additional targeted therapy, hormonal therapy, or radiation therapy is necessary. After completing NAC, patients commonly undergo surgery, and the effectiveness of the treatment is evaluated pathologically in surgical specimens [6]. Different pathological methods are used to assess responses after NAC, with residual cancer burden (RCB) being one of the most comprehensive methods, as it assesses and quantifies the extent of residual disease in the mammary gland and lymph nodes. RCB is calculated as a continuous variable defining four classes, ranging from a complete pathological response (pCR or RCB-0) to chemoresistance (RCB-III) [7].

Chemoresistance, most often detected at the end of NAC, continues to be a great challenge [8], especially in the luminal breast cancer subtype. Although this subtype represents most breast tumors and has the best long-term survival, it is also more likely to exhibit resistance to NAC [9,10,11]. Thus, the clinical decision to administer NAC in this subtype remains controversial [12]. Identifying the underlying mechanisms associated with NAC chemoresistance could enable the application of effective therapies and minimize the potential adverse effects of chemotherapy. Chemoresistance in breast cancer has been attributed to several molecular mechanisms, such as efflux transporters, signaling pathways, non-coding RNAs, and cancer stem cells [13]. While significant progress has been made in unraveling these mechanisms, a comprehensive understanding of the molecular complexities in breast cancer remains a challenging task.

**Scheme 1:**
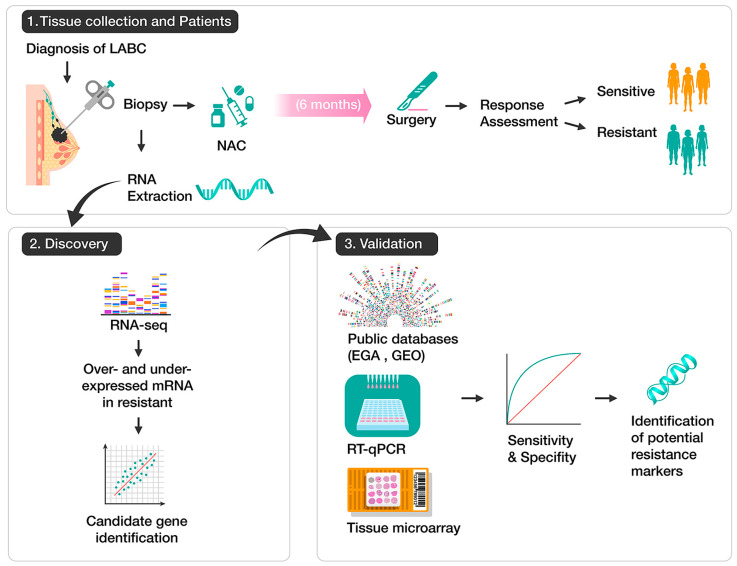
Method used to identify resistance markers in luminal breast cancer. We divided this method into three steps: (1) patients and tissue collection, (2) discovery, and (3) validation.

In the present study, our objective was to search for transcripts via RNA sequencing, which could be associated with chemoresistance in luminal breast cancer, and to evaluate them at the protein level using immunohistochemistry (IHC) to obtain insights into the mechanisms underlying drug resistance that could guide the development of effective treatment strategies. For that purpose, we sequenced the RNA of twelve patients from the National Cancer Institute of Mexico and obtained differentially expressed genes (DEGs) between sensitive and resistant patients (Scheme 1). Subsequently, we evaluated the DEGs that were most associated with resistance and then validated two candidate genes using IHC. We found that the genes solute carrier family 12 member 1 (*SLC12A1*) and glutamate ionotropic receptor AMPA type subunit 4 (*GRIA4*) are associated with NAC resistance in luminal breast cancer.

## 2. Results

### 2.1. Clinicopathological Features in the Breast Cancer Cohort

The description in Table 1 shows the clinicopathological characteristics of the luminal LABC patients included in this study and the associated response to NAC, classified as sensitive or resistant. The response correlated with the clinical stage (*p* = 0.029), in which resistant patients were diagnosed with more advanced clinical stages compared to those who were sensitive. In addition, most resistant patients were estrogen receptor positive (*p* = 0.007) and HER2 negative (*p* = 0.002).

### 2.2. Downregulated Genes in Resistant Patients Were Associated with Pathways Involved in Cancer and Membrane Transport

**Figure 1:**
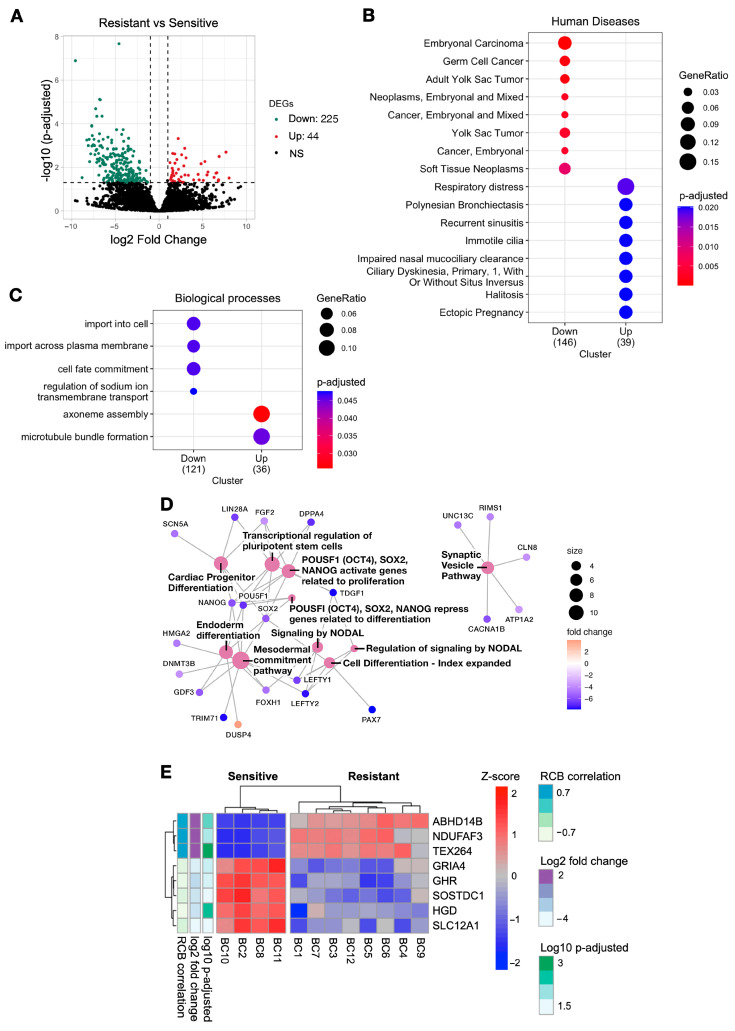
Analysis of differentially expressed genes and functional enrichment in luminal chemoresistant breast cancer patients. (**A**) Volcano plot of the up- and downregulated genes in chemoresistant patients (*p*-adjusted < 0.05, log2 fold change > 1 for upregulation and <1 for downregulation); Genes that did not meet the significance threshold are denoted as ‘NS’; (**B**) Disease enrichment analysis dot plot generated using the DOSE package in R, based on differential expression analysis with *p*-adjusted < 0.05 and log2 fold change ≤ −1 or ≥1. Dot color indicates the *p*-adjusted value of each term, and gene ratio indicates the proportion of genes in the term relative to the total number of genes in the dataset. The plot shows the top 16 most significant human diseases, revealing a significant enrichment of neoplasms in the downregulated genes. (**C**) Gene ontology analysis dot plot of enriched biological concepts, analyzed using the clusterProfiler package in R. The downregulated genes are associated with processes such as import and transport across the cell membrane, while the upregulated genes are involved in the formation and stabilization of microtubules; (**D**) Network of gene and biological concept linkages enriched in resistant patients, based on ConsensusPathDB pathways. Node size reflects the number of genes associated with each biological concept, and edges represent functional relationships between these genes; (**E**) Heatmap of differentially expressed genes that correlate with the residual cancer burden (RCB) classification (correlation ≥ 7 or ≤−7, log2 fold change ≥ 2 or ≤−2, and *p*-adjusted < 0.05). Rows represent genes, and columns represent tissue samples, with expression levels indicated by a color scale ranging from blue (downregulated) to red (upregulated).

With the aim of identifying the genes potentially associated with NAC resistance, we performed whole-transcriptome sequencing on 12 patient samples, comprising 4 sensitive and 8 resistant cases. A differential expression analysis among both groups was performed, obtaining 269 DEGs, most of which were downregulated in resistant patients (Figure 1A). An enrichment analysis was carried out to explore the biological functions and pathways of the 269 DEGs. In Figure 1B, the enriched human diseases are shown. Interestingly, most of the downregulated genes were involved in carcinogenic processes, while upregulated genes were linked with other diseases, such as respiratory diseases. The gene ontology for biological processes is indicated in Figure 1C, where downregulated genes participated in biological processes such as import and transport across the cell membrane, while upregulated genes were involved in the formation and stabilization of microtubules. Furthermore, the participation of the genes in molecular pathways was explored, and the top 10 enriched pathways are shown in Figure 1D. The results revealed a prominent participation of downregulated genes, such as *NODAL*, *POU5F1*, *SOX2*, and *NANOG*, which are related to proliferation and differentiation.

In order to obtain a list of genes that correlate with a response to NAC and could be further validated in this study, a correlation analysis was performed between the DEGs and the RCB. A list of eight genes was retrieved, of which three were upregulated (*ABHD14B*, *NDUFAF3*, *TEX264*) and five were downregulated (*GRIA4*, *GHD*, *SOSTDC1*, *HGD, SLC12A1*) in resistant patients (RCB ≤ −0.7 or ≥0.7, Log2 FC ≤ 2 or ≥2 and *p*-adjusted < 0.05; Figure 1E).

### 2.3. Validation of the DEGs in Breast Cancer Patients

**Figure 2:**
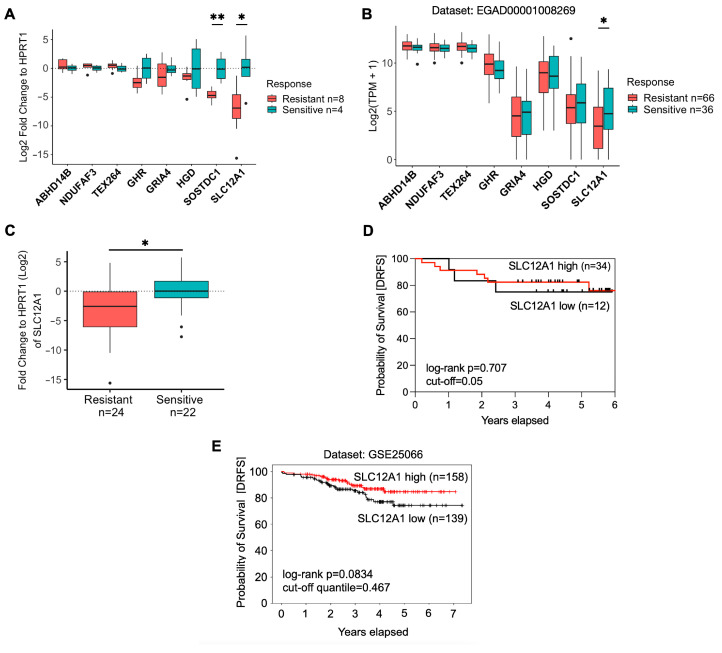
Chemoresistant patients showed downregulation of the solute carrier family 12 member 1 (*SLC12A1*) gene. (**A**) Box plots showing the validation of eight resistance-correlated genes in 12 patients using real-time PCR. Outlying data points are considered as outliers. (**B**) box plots showing gene transcripts per million (TPM) of eight resistance-correlated genes in 102 breast cancer samples from dataset EGAD00001008269 (published by Sammut et al. [14]); (**C**) box plot showing *SLC12A1* mRNA levels in 46 breast cancer samples, measured using real-time PCR; (**D**) Kaplan–Meier graph for distant relapse-free survival (DRFS) of luminal-ER+ patients with low or high expression of *SLC12A1* determined using real-time PCR; (**E**) Kaplan–Meier graph for DRFS of luminal-ER+ breast cancer patients with low or high *SLC12A1* expression from the microarray dataset GSE25066 (published by Hatzis et al. [15]). Median ± range are shown in box plots, and the Mann–Whitney test was used to analyze the data. * *p* < 0.05, ** *p* < 0.005 were considered significant.

The eight correlated genes were validated using real-time PCR and an external RNA-seq dataset (Dataset ID: EGAD00001008269) obtained from the TransNEO study (See Section 4). SOSTDC1 and *SLC12A1* were significantly downregulated in resistant patients using real-time PCR (*p* = 0.004 and *p* = 0.028, respectively; Figure 2A), whereas *SLC12A1* remained the only statistically significant gene in the RNA-seq dataset (*p* = 0.018; Figure 2B).

### 2.4. SLC12A1 Is Associated with Chemosensitivity to NAC

The sample size was increased to validate *SLC12A1* expression using qPCR (*n* = 46, Figure 2C) The analysis revealed a significant decrease in expression among the patients resistant to treatment (*p* = 0.0261). Consequently, we decided to further investigate by examining the presence of the *SLC12A1* protein, also known as the Sodium-Potassium-Chloride Cotransporter 2 or NKCC2. This assessment was conducted using tissue microarrays and involved 31 breast cancer tissue samples collected prior to the start of chemotherapy. At the protein level, NKCC2 was not detected in the breast cancer tissue samples from resistant nor sensitive patients, as shown in Figure S1.

The prognostic significance of *SLC12A1* mRNA was evaluated using the Kaplan–Meier method from the qPCR data. The log-rank test was utilized to compare differences between groups of patients with low and high expression of this gene. The survival analysis was not significant for distant relapse-free survival in our cohort (HR = 0.76 [0.18–3.20]; *p* = 0.707; Figure 2D), suggesting that this mRNA was not a prognostic factor in this cohort of luminal breast cancer patients. Expanding our analysis, we studied a larger external breast cancer dataset (GSE25066 from GEO repository, Figure 2E). Although a trend towards worse distant relapse-free survival (DRFS) was observed in patients with higher gene expression, it lacked statistical significance (HR = 0.58 [0.32–1.08]; *p* = 0.083). Consequently, *SLC12A1* mRNA’s role as a prognostic biomarker in these cohorts seems uncertain. Unfortunately, we could not evaluate overall survival due to limited events in our cohort and the absence of this endpoint in the external database.

The relationship between *SLC12A1* expression and resistance in breast cancer patients was further investigated using bivariate and multivariate logistic regression analyses (Table 2). These analyses, using real-time PCR data, revealed that high expression levels of *SLC12A1* were positively associated with menopausal status (OR = 4.36 [1.13–19.42], *p* = 0.039) and an age of over 50 years (OR = 4.50 [1.12–23.07], *p* = 0.045), but not with other variables such as tumor size, clinical stage, or molecular subtype. The multivariate analysis, adjusted for age and menopausal status, showed that only resistance remained a predictive variable for high expression of *SLC12A1* (OR = 0.09 [0.01–0.48], *p* = 0.011), indicating that the resistant patients were 87% less likely to have high expression of *SLC12A1* compared to the non-resistant patients. The predictive capacity of *SLC12A1* expression to differentiate between the sensitive and resistant patients was also assessed. A receiver operating characteristic (ROC) curve was constructed, where *SLC12A1* showed a sensitivity of 41.67%, specificity of 90.91%, and an area under the curve (AUC) of 0.70, indicating its ability to identify true sensitive patients who benefit from NAC (Table 3). These findings suggest that high expression levels of *SLC12A1* are associated with sensitivity to neoadjuvant chemotherapy in luminal breast cancer patients.

### 2.5. High Levels of GLUR4 Are Associated with Chemoresistance and Worse Prognoses

Ion channels and membrane transporters are well-known to be involved in drug resistance [16,17]. Given *SLC12A*’s known function as an ion transporter and its previously observed correlation with chemoresistance, we investigated *GRIA4*, which encodes the ion channel glutamate receptor 4 (GLUR4). *GRIA4* was chosen from a list of eight correlated DEGs with chemoresistance (Figure 1E), owing to its functional resemblance to *SLC12A1* and existing evidence of its involvement in cancer [18,19,20]. We hypothesized that both genes might collectively contribute to ion transport mechanisms related to chemoresistance in breast cancer.

**Figure 3:**
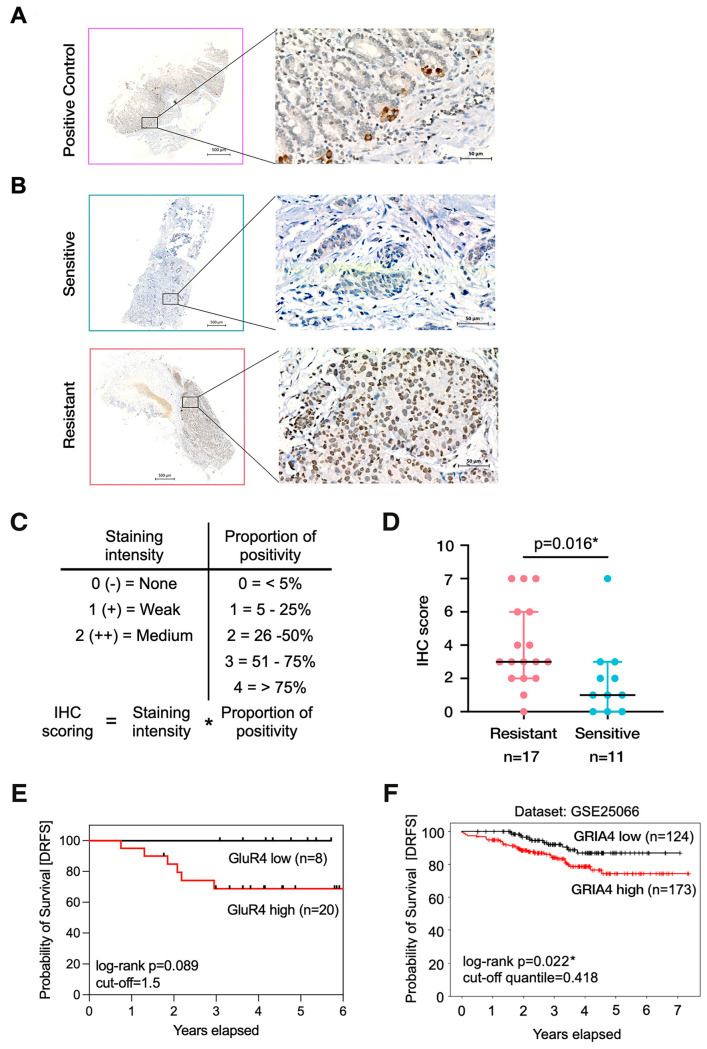
Higher levels of glutamate ionotropic AMPA type subunit 4 (*GRIA4*) protein, GLUR4, were associated with worse outcomes in chemoresistant luminal breast cancer patients. (**A**) Detection of GLUR4 through immunohistochemistry (IHC) in normal colon tissue, utilized as a positive control. Representative magnified image highlights staining along the epithelial cells. Scale bars: 500 μm and 50 μm (zoomed in); (**B**) Representative images of tru-cut biopsies taken before neoadjuvant chemotherapy from breast cancer patients, illustrating the presence of GLUR4 staining. The magnified view highlights the staining within tumor cells. (**C**) Calculation of IHC scores by multiplying staining intensity with the proportion of positivity. (**D**) Scatter plot of IHC scores demonstrating a significant elevation in GLUR4 protein levels in chemoresistant tissues compared to chemosensitive tissues. Data presented as median ± range and analyzed using the Mann–Whitney test. (**E**) Distant relapse-free survival analysis (DRFS) of breast cancer patients based on low or high GLUR4 protein levels from tissue microarrays. (**F**) DRFS analysis of luminal breast cancer patients based on low or high GRIA4 gene expression from microarray dataset GSE25066. * *p* < 0.05 was considered statistically significant.

In our analyses, no significant differences were observed in *GRIA4* mRNA levels between the sensitive and resistant groups. Consequently, we opted to examine *GRIA4* at the protein level (known as GLUR4) in breast cancer patient biopsies obtained before NAC using tissue microarrays, as outlined in Section 4. We found that GLUR4 was significantly increased in resistant patients (*p* = 0.016; Figure 3B,C). In addition, patients with high protein abundance had worse distant relapse-free survival rates, although this was not statistically significant (HR = 4.42 [0.80–24.47]; *p* = 0.089) compared to patients with low protein levels, as shown in the Kaplan–Meyer curve in Figure 3E. To further investigate the prognostic value, *GRIA4* mRNA expression was evaluated in a larger external cohort from the GSE25066 dataset (*n* = 297; Figure 3F). In this cohort, no significant differences in *GRIA4* mRNA levels were found among the sensitive and resistant patients (Figure S2B); however, high mRNA levels were significantly associated with worse prognoses (HR = 2.20 [1.10–4.37]; *p* = 0.022; Figure 3F).

### 2.6. GLUR4 Is a Potential Predictive Marker of Resistance to NAC in Breast Cancer

Our analysis of the IHC data using bivariate logistic regression revealed that a high protein abundance of GLUR4 is an independent predictor of response (OR = 9.00 [1.53–77.22], *p* = 0.023; see Table 4). Moreover, ROC curve analysis demonstrated that GLUR4 protein abundance could effectively identify the patients who were truly resistant to NAC, with GLUR4 showing a sensitivity of 88.24%, specificity of 54.55%, and an AUC of 0.77 (Table 3). These findings suggest that increased levels of GLUR4 protein could be indicative of chemoresistance in luminal LABC. This potential association presents a novel opportunity for developing targeted therapies to address and overcome resistance.

## 3. Discussion

Resistance to NAC is commonly observed in Luminal A subtype tumors, which are defined by their ER+/HER2- characteristics [21]. Our study supports this observation and further demonstrates a significant correlation between NAC resistance and advanced clinical stages, as shown in Table 1.

We utilized RNA-seq to identify potential biomarkers linked to NAC resistance in 12 locally advanced luminal-like breast tumors. The selection for RNA sequencing was based on high RNA integrity numbers (>8.0) and budget considerations. Overcoming this limitation through future collaborative endeavors could enable the analysis of a more extensive sample pool.

Our RNA-seq analysis identified 269 DEGs, with the majority (83.6%) showing downregulation in resistant patients. These DEGs were linked to membrane transport processes, deregulating pathways, and stemness-associated genes, including *NODAL* [22], *POU5F* [23], *SOX2* [24], and *NANOG* [25]. These genes are known contributors to chemoresistance, particularly through ATP-binding cassette (ABC) transporters—a widely recognized mechanism of multidrug resistance [26]. We found eight DEGs that were highly correlated with RCB and then validated them using qPCR and an external database. We focused on the gene *SLC12A1*, because it was the only one that was significantly differentially expressed in both validation databases. We also selected *GRIA4*, because, similar to *SLC12A1*, it is associated with the transport of ions across cell membranes.

*SLC12A1* encodes a sodium–potassium–chloride co-transporter, known as NKCC2, which belongs to the solute carrier (SLC) 12 family of transporters and mediates NaCl reabsorption in the kidney [27]. *SLC12A1* has been implicated in tumors, acting as an oncogene in hepatocellular carcinoma [28] or as a tumor suppressor in renal cell carcinoma [29]. It has also been suggested to play a role in chemoresistance in ovarian cancer [30]. SLC transporter members are associated with prognosis in breast cancer [31,32,33] and chemoresistance in tumors [30,34,35]. For instance, a study found that the organic cation transporter *SLC22A16* mediates doxorubicin influx and conferred sensitivity in leukemic cells [36]. In another study, *SLC22A16* upregulation was associated with poor overall survival in gastric cancer [37]. For these reasons, SLC transporters significantly impact cancer biology and chemoresistance.

In our study of breast cancer patients, we observed a decrease in *SLC12A1* expression among the resistant patients compared to the sensitive patients. This finding suggests a potential role for *SLC12A1* in mediating drug influx in cancer cells, with its downregulation possibly conferring resistance to chemotherapy, similar to what has been observed with other SLC transporters such as *SLC22A16* [36]. However, we were unable to detect this change in expression at the protein level in the tissues of either sensitive or resistant patients, possibly due to the sensitivity of the test and/or the small number of samples analyzed. The limited sample size may have contributed to the lack of significant statistical results for *SLC12A1* in Figure 2D,E. In addition, due to the limited amount of tissue obtained from the tru-cut biopsies, we were unable to definitively validate the specificity of the polyclonal antibodies used in our experiments using other techniques, such as Western blotting or ELISAs. However, our experiments included positive controls that demonstrated specificity in the evaluated tissues, as shown in Figure S1A and Figure 3B. Despite these limitations, our findings provide valuable insights into the potential role of *SLC12A1* mRNA expression in the response to NAC in breast cancer. To the best of our knowledge, this is the first study in which a member of the SLC12 family of inorganic ion transporters is potentially associated with chemoresistance in breast cancer, giving insights into another possible mechanism underlying drug resistance.

Building upon our previous results, which showed a significant downregulation of genes involved in import and transmembrane transport (Figure 1C) and a strong association between the ion transporter *SLC12A1* and chemoresistance (Figure 2A–C), we further investigated another gene, *GRIA4*, which is involved in ion transport and was found to be differentially expressed in the RNA-seq analysis. *GRIA4* encodes a subunit of the AMPA tetrameric receptor complex, known as GLUR4, which belongs to the ionotropic glutamate receptors (iGluRs). iGluRs are quaternary ligand-gated ion channels that allow cation influx upon glutamate binding and play an important role in the synaptic transition in the central nervous system [38]. Interestingly, iGluRs have been involved in chemotherapy response across various cancers. They have been found to be upregulated in hepatocellular carcinoma [39], as well as in chemosensitive ovarian serous adenocarcinoma [40] and chemoresistant glioma [41].

In our validation analyses of the breast cancer cohorts, we did not observe significant differences in *GRIA4* transcript levels between the sensitive and resistant patients. However, we found that higher GLUR4 protein levels correlated with a worse prognosis and were more prevalent in the resistant patients. Interestingly, similar contrasting results regarding *GRIA4* mRNA and protein levels have been reported in other studies conducted in colorectal cancer [20,42]. These studies propose that post-transcriptional mechanisms and variations in the half-lives of *GRIA4* transcript variants might potentially explain this phenomenon. Although our study did not specifically investigate different *GRIA4* isoforms, it is worth highlighting that the antibody employed in our research detects both long and short isoforms. To achieve a more comprehensive understanding of *GRIA4*’s role in breast cancer biology and its potential connection to chemoresistance, it will be crucial to explore these isoforms in future investigations.

In conclusion, our study revealed that breast cancer patients resistant to chemotherapy had decreased levels of *SLC12A1* mRNA and increased levels of GLUR4 protein. Our multivariate analysis showed that *SLC12A1* expression was significantly associated with age, menopause, and chemoresistance, while GLUR4 correlated with chemoresistance and predicted worse outcomes for breast cancer patients. These findings suggest that *SLC12A1* and GLUR4 may play a crucial role in breast cancer chemoresistance, as they are involved in ion transport into cells. However, additional studies involving larger patient cohorts are necessary to validate our findings, confirm the accuracy of the proposed potential biomarkers, and elucidate the molecular mechanisms underlying resistance. This will also help to establish whether *SLC12A1* and GLUR4 could serve as potential clinically relevant targets for therapeutic interventions across diverse breast cancer subtypes. Understanding the roles of ion transporters and channels is crucial to gaining insight into drug resistance mechanisms and developing new treatment strategies. Our study highlights the potential for *SLC12A1* and GLUR4 to be used as therapeutic targets in breast cancer patients, providing valuable insights into the mechanisms underlying chemoresistance. By targeting these transporters and channels, novel treatment approaches could be developed that may improve patient outcomes.

## 4. Materials and Methods

### 4.1. Patients and Collection of Tissue Samples

In this study, we performed RNA-seq analysis on RNA samples obtained from 12 female patients with luminal breast cancer (Table S1). The DEGs identified in these samples were validated in a larger cohort of 62 patients, which included the initial 12 RNA samples. All the patients were female, with ages ranging from 34 to 68 years, and were with LABC at stages IIB to IIIC at the National Cancer Institute of Mexico between January 2012 and December 2015. Furthermore, all the patients had hormone receptor-positive tumors confirmed by an estrogen receptor-positive (ER+) and/or progesterone receptor-positive (PR+) status. The patients were further classified into subtypes based on their receptor status, including luminal A (ER/PR+, HER2-, KI67 < 20%), luminal B HER2- (ER/PR+, HER2-, KI67 ≥ 20%), or luminal B HER2+ (ER/PR+, HER2+, any KI67%).

Tissue samples were collected prior to treatment, with all the patients receiving an NAC regimen of anthracyclines and taxanes in a sequential schedule, as described in a previous study by Contreras-Espinosa L. et al. [43]. After NAC completion, the patients underwent surgery, and the response to NAC was assessed by a pathologist in the surgical resection specimens. The response was stratified according to the RCB [7] using the MD Anderson Cancer Center’s online calculator (http://www3.mdanderson.org/app/medcalc/index.cfm?pagename=jsconvert3 accessed on 1 December 2017). Chemosensitive patients were classified as RCB-0/I, while non-responders or chemoresistant patients were classified as RCB-II/III. Informed consent was obtained, and this study was approved by the ethical and research committee of the National Cancer Institute of Mexico (018055DII CEI130218).

### 4.2. RNA Extraction and RT-qPCR

Total RNA extraction from the patient samples was performed using the AllPrep kit (QIAGEN, Germantown, MD, USA), and the RNA concentration and quality analysis (RIN value) were evaluated using a Tape Station 2200 bioanalyzer (Agilent Technologies, Santa Clara, CA, USA). Subsequently, 300 ng of RNA was transcribed into cDNA using the High-Capacity cDNA Reverse Transcription Kit (Thermo Fisher Scientific, Waltham, MA, USA), and real-time PCR was carried out using SYBR Green/ROX qPCR master mix (2×) (Thermo Fisher Scientific, Waltham, MA, USA) on a QuantStudio 3 real-time PCR system (Thermo Fisher Scientific, Waltham, MA, USA). The levels of gene expression were measured using the 2^−ΔΔCt^ method [44]. Ct values were normalized to the expression levels of *HPRT1* as a housekeeping gene and then subtracted from the expression of the sensitive patients as a reference group. The following primers were used:

Forward primer (*HPRT1*): 5′-AGGGTGTTTATTCCTCATGG-3′;

Reverse primer (*HPRT1*): 5′-CACAGAGGGCTACAATGTG-3′.

Forward primer (*SLC12A1*): 5′-CTTGCTGGTGCCAATATCTC-3′;

Reverse primer (*SLC12A1*): 5′-CTAAGTAGGCAACAGTGGTG-3′.

Forward primer (*GRIA4*): 5′-CTCATCACACCAAGTTTCCC-3′;

Reverse primer (*GRIA4*): 5′-CGTAGTGATCCAGCAAACTC-3′.

### 4.3. RNA-Seq and Data Analysis

Total RNA sequencing was performed as described in a previous study [43]. Twelve patients (four sensitive and eight resistant) were selected for RNA sequencing. The RNA-seq data are available from the NCBI Gene Expression Omnibus (GEO) at https://www.ncbi.nlm.nih.gov/geo/ (accessed on 5 September 2018), accession number GSE159448 [43].

Sequence quality was checked in FastQC (v0.11.7), and data filtering and adapter trimming were performed using Trimmomatic (v0.38). Subsequently, the data were pseudo-aligned to the reference transcriptome using the Salmon software [45] (v0.11.3). Reference sequences and annotations were downloaded from GENECODE (v31) from the ENSEMBL website (UCSC, NCBI, ENSEMBL). PCA analysis was performed to check for bias and covariates. A batch effect was detected for ER status and was added as a covariate in the DEseq2 design (~ER status + response). To obtain DEGs between the sensitive and resistant patients, DEseq2 [46] (v1.24) from the Bioconductor package was used. A gene set analysis using the clusterProfiler and DOSE packages was performed to explore the role of the DEGs across human diseases, biological processes, and molecular pathways within ConsensusPathDB [47]. *p* < 0.05 was set as the cut-off for the minimum overlap criterion. To obtain the genes most significantly associated with resistance for further validation, extra filtering of the genes was implemented using a Pearson correlation analysis with the RCB classes, considering those with an absolute value of seven to be highly correlated. A *p*-adjusted threshold of <0.05 and log2 fold change < −1.5 or >1.5 were set to detect significantly expressed genes.

### 4.4. External Breast Cancer Datasets

The expressions of the genes that correlated with RCB were validated in a dataset containing 336 patients enrolled in the TransNEO cohort from the University of Cambridge, UK [14]. The data were deposited at the European Genome–Phenome Archive under accession number EGAS00001004582 (Dataset ID: EGAD00001008269). This study only recorded the status of estrogen receptors, excluding progesterone receptors. Therefore, for our analysis, we included 102 ER+ patients for whom there were RNA-seq data and who received NAC (66 resistant and 36 sensitive). To identify a set of upregulated or downregulated genes in the resistant patients, a differential expression analysis was performed on the gene raw counts using the DESeq2 package (v1.24).

The prognostic significance of the biomarkers was assessed in a second external dataset, as the TransNEO dataset had no record of the prognosis. A dataset comprising 508 patients from different cohorts [15] was used to assess survival. This dataset is available via the GEO repository (http://www.ncbi.nlm.nih.gov/geo/ accessed on 2 June 2021) with accession ID: GSE25066. Distant relapse-free survival (DRFS) for high or low levels of gene expression was estimated from 297 selected ER+ patients using the Cancer Target Gene Screening (CTGS) web application, available at http://ctgs.biohackers.net/GSE25066/survival-quantities/ (accessed on 1 April 2021).

### 4.5. Tissue Microarrays and Immunohistochemical Assay

A tissue microarray was constructed to analyze the presence of NKCC2 and GLUR4 proteins in breast cancer tissues using IHC. First, a pathologist selected the tumoral area from FFPE tissue samples obtained from core needle biopsies prior to chemotherapy. Then, the extracted tissue cores were re-embedded into a recipient block (microarray). Paraffin blocks from the tissue microarrays were cut into 5 μm slices. Positive controls from adjacent healthy tumor tissue were included in each TMA, displaying 13 cases of sensitive breast cancer patients and 18 cases of resistant breast cancer patients.

The primary antibody for *SLC12A1* was purchased from Sigma-Aldrich (St. Louis, MO, USA), specified as a rabbit polyclonal, anti-human antibody under catalog number HPA018107. The primary antibody for *GRIA4* was purchased from Thermo Fisher Scientific (Waltham, MA, USA), identified as a rabbit polyclonal, anti-human antibody with catalog number PA5-24217. The IHC staining was performed using a Ventana BenchMark LT automated immunostainer (Roche Diagnostics, Indianapolis, IN, USA) following the manufacturer’s instructions. The slides were counter-stained with hematoxylin and mounted in non-aqueous mounting media. The slides were then imaged using an AxioScan.Z1 microscope and the ZEN 2.6 Slidescan software (Zeiss AG, Oberkochen, Germany), using an objective Plan-Apochromat 20×/0.8 M27. The images were magnified to 20× and 400×. An experienced pathologist evaluated the images, providing the percentage of tumor cells with at least 1% staining and the intensity of staining as 0 (−), none; 1 (+), weak; and 2 (++), medium. The data provided by the pathologist were used to derive an overall IHC staining score criterion by multiplying the scores for the intensity and percentage of tumor cells stained, as described in a previous study by Wang et al. [48].

### 4.6. Statistical Analyses

All the analyses were performed using the R software (v4.2.1). The baseline characteristics of the included breast cancer patients are presented as numbers (percentages) for nominal variables and means (ranges) for continuous variables. To determine the association between the clinicopathologic data and the response, the Mann–Whitney U test was used for continuous variables, and Yate’s Chi-squared test was used for nominal variables. The distribution of the RT-qPCR and IHC data was explored using the Shapiro–Wilk and Kolmogorov–Smirnov tests. Because the data did not present a normal distribution, the Mann–Whitney U test for non-parametric data was used to determine whether the medians of the sensitive and resistant data were equal.

To evaluate the performance of the chemoresistance-associated genes as potential predictive markers, the sensitivity and specificity were determined using ROC curves. A cut-off point was selected from the ROC curves, which could predict the patients with the worst prognosis. The Kaplan–Meier method was employed for a survival analysis of DRFS, measured from the start of the NAC treatment to distant recurrence or death from any cause within a 6-year period. The log-rank test was used for statistical analysis. Patients without an event were censored at the date of last follow-up. *p* < 0.05 was considered statistically significant for all the tests.

## Figures and Tables

**Table 1:** Baseline characteristics of chemosensitive and chemoresistant luminal breast cancer patients included in this study (*n* = 62).

Variable	Chemosensitive (*n* = 28)	Chemoresistant (*n* = 34)	*p*-Value
**Age, yrs**			
Mean (range)	49.7 (34–68)	50.7 (34–68)	0.865 ^a^
**BMI, kg/m^2^**			
Mean (range)	29.2 (19.7–40.2)	29.5 (22.7–42.6)	0.932 ^a^
**Tumor size, cm**			
Mean (range)	6.6 (3–15)	6.4 (1.5–14)	0.691 ^a^
**Menopausal status**			
Pre	13 (46.4%)	15 (44.1%)	1 ^b^
Post	15 (53.6%)	19 (55.9%)	
**Histological subtype**			
Ductal	25 (89.3%)	31 (91.2%)	1 ^b^
Lobular	3 (10.7%)	3 (8.8%)	
**Clinical stage**			
II	8 (28.6%)	3 (8.8%)	0.029 ^b,^*
III	20 (71.4%)	31 (91.2%)	
**Grade**			
Low	4 (14.3%)	4 (11.7%)	0.249 ^b^
Intermediate	9 (32.1%)	19 (55.9%)	
High	15 (53.6%)	12 (35.3%)	
**KI67**			
Low (<20%)	2 (7.1%)	11 (32.4%)	0.089 ^b^
High (≥20%)	26 (92.9%)	23 (67.6%)	
**ER status**			
Positive	21 (75%)	34 (100%)	0.007 ^b,^*
Negative	7 (25%)	0 (0%)	
**PR status**			
Positive	33 (75%)	34 (100%)	0.389 ^b^
Negative	2 (25%)	0 (0%)	
**HER2 status**			
Positive	13 (46.4%)	3 (8.8%)	0.002 ^b,^*
Negative	15 (53.6%)	31 (91.2%)	
**Subtype**			
Luminal A	2 (7.1%)	11 (32.4%)	0.001 ^b,^*
Luminal B HER2-	13 (46.4%)	20 (58.8%)	
Luminal B HER2+	13 (46.4%)	3 (8.8%)	
**Recurrence**			
Yes	4 (14.3%)	9 (26.5%)	0.39 ^b^
No	24 (85.7%)	25 (73.5%)	
**Status**			
Death	2 (7.1%)	4 (11.8%)	0.856 ^b^
Alive	26 (92.9%)	30 (88.2%)	

^a^ Mann–Whitney U test. ^b^ Yates’ Chi-squared test. * *p* < 0.05. Abbreviations: BMI, body mass index; ER, estrogen receptor; HER2, human epidermal growth factor receptor 2.

**Table 2:** Bivariate and multivariate logistic regression analysis to identify the variables related to *SLC12A1* expression (high vs. low, cutoff = 0.05) in patients resistant to neoadjuvant chemotherapy (*n* = 46).

	Bivariate			Multivariate		
	OR	95% CI	*p*-Value	OR	95% CI	*p*-Value
**Age (≥50 vs. <50)**	4.50	[1.12–23.07]	0.045 *	6.06	[0.20–133.15]	0.243
**Menopausal status (positive vs. negative)**	4.36	[1.13–19.42]	0.039 *	1.22	[0.07–34.70]	0.891
**Tumor size (T3/T4 vs. T1/T2)**	1.25	[0.28–4.99]	0.756			
**Clinical stage (III vs. II)**	0.80	[0.20–3.57]	0.756			
**Luminal A (positive vs. negative)**	0.47	[0.07–3.94]	0.440			
**Luminal B HER2- (positive vs. negative)**	0.40	[0.08–1.59]	0.215			
**Luminal B HER2+ (positive vs. negative)**	6.5	[1.07–125.78]	0.089			
**Resistance (positive vs. negative)**	0.13	[0.02–0.60]	0.017 *	0.09	[0.01–0.48]	0.011 *

* *p* < 0.05. Abbreviations: OR, odds-ratio; CI, confidence interval.

**Table 3:** Predictive performance of marker candidates for neoadjuvant chemotherapy response in luminal breast cancer patients.

Biomarker	Detection Method	AUC (95% Cl)	*p*-Value	Cut-Off	Sensitivity (95% Cl)	Specificity (95% Cl)
**SLC12A1**	Real-time PCR	0.70 (0.54–0.85)	0.026	0.05	41.67 (24.47–61.17)	90.91 (72.19–98.38)
**GLUR4**	IHC	0.77 (0.58–0.96)	0.019	1.5	88.24 (65.66–97.91)	54.55 (28.01–78.73)

Abbreviations: *SLC12A1*, solute carrier family 12 member 1; GLUR4, glutamate ionotropic AMPA type subunit 4; AUC, area under the curve; CI, confidence interval; PCR, polymerase chain reaction; IHC, immunohistochemistry.

**Table 4:** Bivariate logistic regression analysis to identify the variables related to GLUR4 protein levels (high vs. low, cutoff = 1.5) of patients resistant to neoadjuvant chemotherapy (*n* = 28).

	OR	95% CI	*p*-Value
**Age (≥50 vs. <50)**	2.46	[0.44–19.66]	0.335
**Tumor size (T3/T4 vs. T1/T2)**	0.21	[0.01–1.55]	0.185
**Lymph nodes (N1, N2, N3 vs. N0)**	11.40	[1.18–260.86]	0.053
**Clinical stage (III vs. II)**	0.57	[0.03–4.81]	0.643
**Luminal A (positive vs. negative)**	0.90	[0.17–5.43]	0.901
**Luminal B HER2- (positive vs. negative)**	1.50	[0.28–8.16]	0.630
**Luminal B HER2+ (positive vs. negative)**	0.37	[0.01–10.16]	0.500
**Resistance (positive vs. negative)**	9.00	[1.53–77.22]	0.023 *

* *p* < 0.05. Abbreviations: OR, odds ratio; CI, confidence interval.

## Data Availability

The data generated in this study are openly available in the NCBI Gene Expression Omnibus (GEO) at https://www.ncbi.nlm.nih.gov/geo/ (accessed on 5 September 2018), accession number GSE159448.

## Supplementary Materials

The supporting information can be downloaded at: https://www.mdpi.com/article/10.3390/ijms242216104/s1.
