# Supplementary material for: Association of SLC12A1 and GLUR4 Ion Transporters with Neoadjuvant Chemoresistance in Luminal Locally Advanced Breast Cancer
Source: Int J Mol Sci. 2023 Nov 9;24(22):16104. doi: 10.3390/ijms242216104 (PMC10670992; doi:10.3390/ijms242216104)
Supplement: Supplementary file 1 [file ijms-24-16104-s001.zip › ijms-2664356-supplementary.pdf]

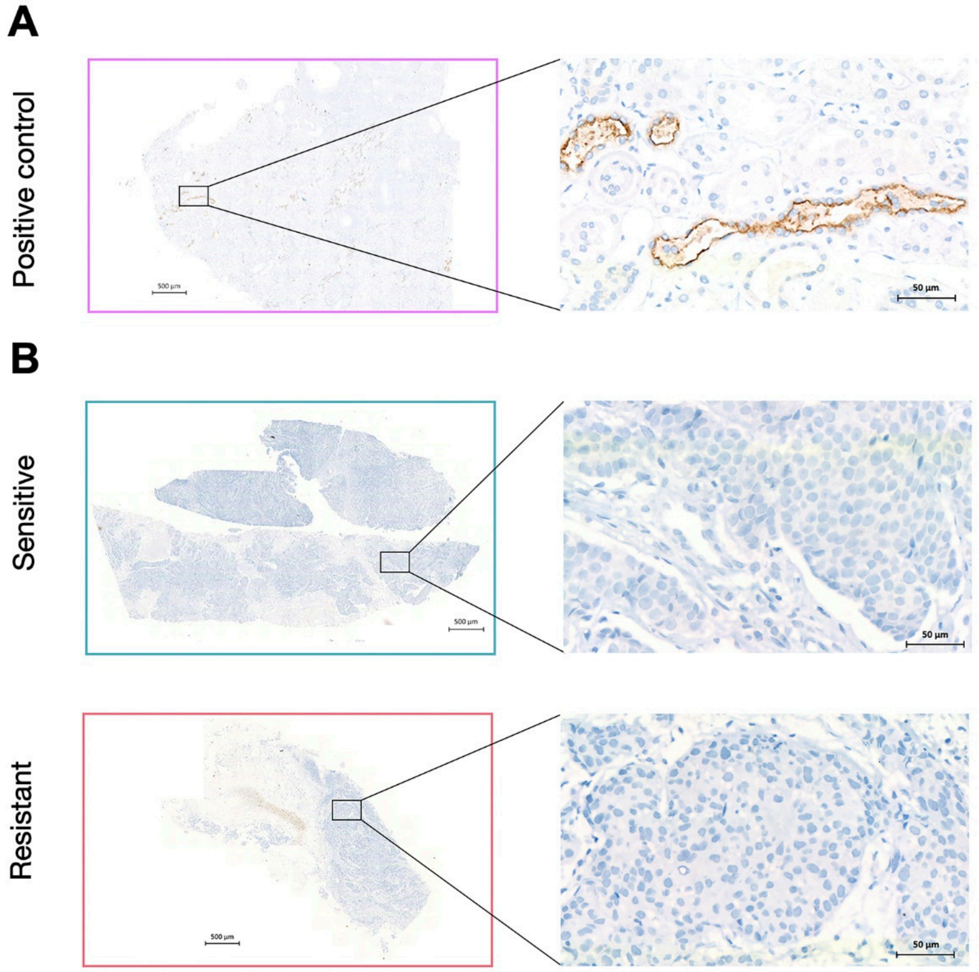

Figure S1. *SLC12A1* protein, NKCC2, was not detected in breast cancer tissues using immunohistochemistry.

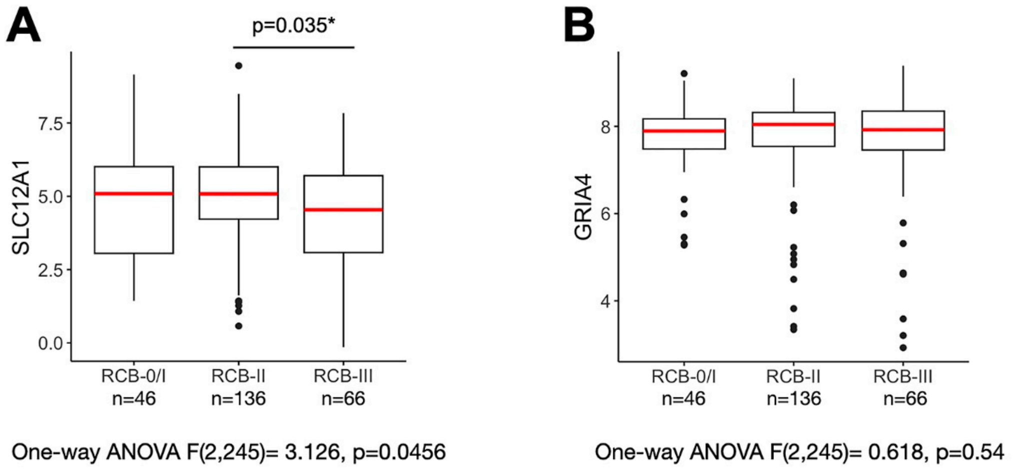

Figure S2. Gene expression evaluation of candidate resistance biomarkers *SLC12A1* and *GRIA4* across the RCB classes from the microarray GSE25066.

Table S1. Baseline characteristics of luminal breast cancer patients included in the RNA sequencing analysis (n=12).

Patient ID	Age	Estrogen Receptor	Progesterone Receptor	HER2	%Ki67	Clinical Stage	Subtype	RCB index	RCB class	Response
BC1	52	Positive	Positive	Negative	60	IIIB	Luminal B HER2-	3.301	RCB-III	Resistant
BC2	66	Positive	Positive	Positive	90	IIB	Luminal B HER2+	3.889	RCB-III	Resistant
BC3	52	Positive	Positive	Negative	18	IIIA	Luminal A	5.752	RCB-III	Resistant
BC4	50	Positive	Positive	Negative	25	IIIA	Luminal B HER2-	2.617	RCB-II	Resistant
BC5	56	Positive	Positive	Negative	35	IIIA	Luminal B HER2-	4.334	RCB-III	Resistant
BC6	42	Positive	Positive	Negative	30	IIB	Luminal B HER2-	3.65	RCB-III	Resistant
BC7	47	Positive	Positive	Negative	80	IIIA	Luminal B HER2-	1.237	RCB-I	Sensitive
BC8	43	Positive	Positive	Negative	10	IIIA	Luminal A	4.199	RCB-III	Resistant
BC9	68	Positive	Positive	Positive	50	IIIA	Luminal B HER2+	0	RCB-0	Sensitive

BC10	48	Negative	Positive	Positive	50	IIIB	Luminal B HER2+	0	RCB-0	Sensitive
BC11	40	Negative	Positive	Negative	60	IIB	Luminal B HER2-	0	RCB-0	Sensitive
BC12	39	Positive	Positive	Positive	50	IIIA	Luminal B HER2+	3.154	RCB-II	Resistant

Abbreviations: HER2, human epidermal growth factor receptor-2; RCB, residual cancer burden.
